# Effectiveness of iodoform-based filling materials in root canal treatment of deciduous teeth: a systematic review and meta-analysis

**DOI:** 10.1080/26415275.2022.2060232

**Published:** 2022-05-19

**Authors:** Manoelito Ferreira Silva Junior, Leticia Maíra Wambier, Mayara Vitorino Gevert, Ana Cláudia Rodrigues Chibinski

**Affiliations:** State Universiy of Ponta Grossa, Avenue General Carlos Cavalcanti, Ponta Grossa, Brazil

**Keywords:** Pulpectomy, tooth, deciduous, dental materials

## Abstract

**Introduction:**

The objective was to review the effectiveness of iodoform-based compared to noniodoform-based filling materials in the root canal treatment of deciduous teeth.

**Methods:**

This systematic review and meta-analysis used randomized clinical trials with six months or more follow-up. The risk of bias of individual studies and the certainty of the evidence were evaluated (Cochrane risk of bias tool and GRADE, respectively).

**Results:**

The initial search resulted in 5,127 studies after removal of duplicates. After screening by title and abstract, 34 full-text studies were eligible and 21 remained in the qualitative synthesis and 19 in the meta-analysis. Iodoform-based filling materials resulted in fewer clinical failures when compared to noniodoform-based filling materials at the 6 months (OR = 0.43, 95%CI: 0.19–0.97, *p* = .04) and 9–12 months (OR = 0.46, 95%CI: 0.23–0.93, *p* = .03), but not at the 18–30 months follow-up (OR = 1.08, 95%CI: 0.58–2.03, *p* = .81). When considering radiographic failures, there was no statistical difference between iodoform-based and noniodoform-based filling materials at the 6 months (OR = 0.72, 95%CI: 0.39–1.32, *p* = .29) and 18–30 months follow-ups (OR = 1.06, 95%CI: 0.51–2.21, *p* = .87), but fewer radiographic failures were detected at the 9–12 months follow-up (OR = 0.49, 95%CI: 0.29–0.80, *p* = .005).

**Conclusion:**

Iodoform-based filling materials showed better clinical and radiographic performance when compared to non-iodoform-based filling materials in the short term, and similar performance in the long term. However, most of the studies exhibited unclear or high risk of bias and the overall certainty of the evidence ranged from low to very low. Therefore, new randomized clinical trials must be accomplished to corroborate this conclusion.

## Introduction

Endodontic treatment with complete pulp removal—pulpectomy—is commonly used in deciduous teeth with irreversible pulpitis or necrotic pulp [1,2]. Usually, after the chemomechanical preparation of the root canals, an absorbable and biocompatible material must be used to favor repair and allow the permanence of the tooth in the mouth till its physiological exfoliation [1]. In an attempt to simplify the steps, Lesion Sterilization and Tissue Repair (LSTR) therapy has been investigated for the treatment of primary tooth canals. This technique implies nonmechanical preparation of the root canals and placement of a paste made of a mixture of antibiotics at the entrance of the root canals [3].

The complex morphology of root canal systems in primary teeth can hamper mechanical instrumentation, irrigation and/or disinfection [4]. For this reason, the use of substances with antimicrobial properties is generally used to increase the chances of a successful endodontic treatment [1]. Even in LSTR therapy, the use of a mixture of antibiotics is considered sufficient to respond to the periapical lesions [3].

A good filling material must be absorbable and able to accompany the deciduous tooth root resorption and not interfere with the germ of the successive permanent tooth [1,2]. For a long time, zinc oxide eugenol (ZOE) was the material most used [4–21] but its use was reduced [22] due to its limited antimicrobial action [22–24], slower resorption compared to that of the deciduous teeth [8,24], and its ability to generate a foreign body-type reaction if the material overflows the root apex [25]. The use of other filling materials has grown, mainly calcium hydroxide with iodoform [4–7,9,10,14–17,19,20,22,23,26–30], since they exhibit pronounced antimicrobial action, are easily absorbed when overflowing the tooth apex [31–33], are radiopaque, and can be purchased as a pre-mixed paste for easy application [4].

An ideal filling material for deciduous teeth has not been found yet [4–7]. However, the use of iodoform in different filling materials stands out due to the resorption capability and good antimicrobial properties [8,10,34]. In the market, there are different formulations with iodoform, including ZOE [9,11–13,16–19,21,35,36], calcium hydroxide [9–12,14–20,22,23,27–29,35], antibiotic agents [37,38], or other substances [39,40].

Previous systematic reviews have focused on different filling materials for deciduous teeth after endodontic treatment [4–7], on the chemomechanical technique or LSTR therapy [3]. However, there is no review investigating possible advantages of iodoform addition to filling materials, independent of the endodontic technique used. The objective this study was to review the effectiveness of iodoform-based compared to non-iodoform-based filling materials in the root canal treatment of deciduous teeth.

## Materials and methods

### Protocol and registration

The study protocol was registered in the PROSPERO database (CRD42019123937), and the recommendations of the Preferred Reporting Items for Systematic Reviews and Meta-Analyses (PRISMA) statement were followed [41]. The study was accomplished from December 2018 to June 2019 and updated in March 2021.

### Information sources and search strategy

- P (Population) = children with deciduous teeth that received root canal treatment;
- I (Intervention) = root canal treatment using iodoform-based filling materials;
- C (Comparison) = root canal treatment using noniodoform-based filling materials;
- O (Outcomes) = clinical and/or radiographic success/failure;
- S (Type of studies) = randomized clinical trials.

The search used the following electronic data bases: MEDLINE *via* PubMeb, Scopus, Web of Science, Latin American and Caribbean Literature on Health Sciences (LILACS), Biblioteca Brasileira em Odontologia (BBO) (Brazilian Dentistry Library) and Cochrane Library.

The search strategy (Supplement 1) was based on controlled vocabulary (MeSH terms) of the PubMed database along with free keywords retrieved from titles and abstracts. MeSH terms and free keywords were initially combined in each item using the Boolean operator ‘OR’. The Population and Intervention were combined to build the search strategy by the Boolean operator ‘AND’. The search strategy developed for PubMed was adapted to other electronic databases. We also hand-searched the reference lists of all primary studies for additional relevant publications.

The grey literature was searched using the databases System for Information on Grey Literature in Europe (SIGLE) and Scholar Google. Dissertations and theses were searched using the ProQuest Dissertations and Theses Full‐Text databases and the Periódicos Capes Thesis database.

### Eligibility criteria

Randomized Clinical Trials (RCT) were included. The studies excluded comprised noncontrolled clinical studies, editorial letters, literature reviews, *in vitro* or animal studies, observational studies, case reports and case series. Studies written in Chinese and Japanese were also excluded. No restrictions on publication dates were applied.

### Study selection and data collection process

The retrieved studies were imported into a reference management software (EndNote X9 - Clarivate Analytics, Philadelphia, USA). After removal of duplicates, studies were excluded after title and abstract reading according to the exclusion criteria previously described. This process was performed independently by two reviewers (M.F.S.J. and A.C.R.C.); in case of disagreement, a third reviewer was consulted (L.M.W.).

The studies were selected by title and abstracts accordingly to the described eligibility criteria. Full-text studies were obtained when there was insufficient information in the title and abstract to make a clear decision.

Eligible studies received an identification that combined the first author’s name and the year of publication. The data from the studies were extracted to customized extraction forms that comprised the study design, participants, interventions and outcomes. Studies reporting different follow-ups of the same research were only considered once to prevent data overlapping. This process was performed independently by two reviewers (M.F.S.J. and A.C.R.C.); in case of disagreement, a third reviewer was consulted (L.M.W.).

### Risk of bias in individual studies

The evaluation of the risk of bias of the selected studies was carried out by two independent reviewers (M.F.S.J. and A.C.R.C.), using the *Cochrane Collaboration* tool to evaluate the risk of bias in RCT [42]. The evaluation criteria comprised five items: (1) sequence generation, (2) allocation concealment, (3) blinding of result evaluators, (4) incomplete result data, and (5) selective result reports. In case of discrepancies between the evaluators, a third reviewer was consulted (L.M.W.).

The risk of bias was rated following the recommendations described in the *Cochrane Handbook for Systematic Reviews of Interventions* 5.1.0. Each criterion was graded as having ‘low’, ‘unclear’ or ‘high’ risk of bias, accordingly to the information retrieved in the text regarding potential bias.

The study was judged as ‘high’ risk if at least one key domain was not achieved adequately. At the study level, a study was judged as having a ‘low’ risk of bias if all domains were considered at ‘low’ risk. If the information could not be retrieved or was incomplete on one of these domains, without presenting a ‘high’ risk of bias in any domain, the study was considered as having an ‘unclear’ risk of bias.

### Meta-analysis

The outcomes assessed were clinical or radiographic failure (yes or no) for the different follow-ups (6, 9–12 and 18–30 months) and techniques for tooth preparation (chemomechanical and LSTR). The results were summarized using the random-effects model to estimate the Odds Ratio (OR) using a 95% confidence interval (95%CI). The heterogeneity was assessed using the Cochran Q test and the I^2^ statistics. All analyses were performed using the software Revman 5 (Review Manager ver. 5, The Cochrane Collaboration, Copenhagen, Denmark).

#### Assessment of the certainty of the evidence

The certainty of the evidence was assessed using the Grading of Recommendations Assessment, Development and Evaluation (GRADE) [43]. This stage was accomplished to determine the overall certainty of the evidence for each meta-analysis. The evidence can be graded in 4 levels (very low, low, moderate, high). When a meta-analysis is graded as ‘high quality’, it means that the authors are very confident that the true effect lies close to the estimate of the effect.

## Results

### Selection of studies

The initial search in the databases resulted in 6,049 registers (Supplement 1). The removal of duplicates resulted in 5,127 registers. After the selection based on title, the number of registers was reduced to 96. A total of 62 registers was excluded after the abstract reading, resulting in 34 full text for the eligibility assessment. Thirteen registers were excluded due to: (1) nonrandomized clinical trial [22] (2) absence of a group using iodoform-based filling material [44,45], (3) all groups used iodoform-based filling materials [34,46–49], (4) original dissertation of an included study [50], (5) publication of preliminary results of an included study [51,52], and (6) text in Chinese [53,54] (Supplement 2).

### Characteristics of the included studies

The characteristics of the 21 selected studies are listed in Table 1. The randomization unit of the clinical studies was either the patient [10–12,20,27,29], or the tooth [9,13–18,21,28,30,36–38]. In some studies, the randomization unit was not identified [19,35].

**Table 1.** Summary of some methodologic characteristics of the included studies.

Author and date	Age: years (average)	Number of sample participants Performing Sample calculation Gender distribution	Number of teeth (Type) Reason for treatment Number of operators (sessions and technique) Number of evaluators (blinding and calibration)	Iodoform-based filling materials (Trademark or Composition)	Noniodoform-based filling materials (Trademark or composition)	Follow-up time (months)	Clinical and/or radiographic evaluation criteria
Al-Ostwani et al., 2016 [9]	3-9.	39. Unreported sample calculation. 19 girls. 20 boys.	64 (molars). Caries. 3 operators (one session and chemical-mechanical technique). 2 evaluators (double-blinded and calibration not reported).	- Metapex (Meta Biomed / Korea). -Endoflas-CF (adding 56.5% zinc oxide, 40.6% iodoform, 1.63% barium sulfate and 1.07% calcium hydroxide, and mixed with eugenol without adding chlorophenol).	- ZOE (Fares, Damascus, Syria, License 0009). - ZOP (50% zinc oxide powder with 50% hydrolytic própolis - Syrian patent number/5918).	6 and 12.	Clinical success: Based on the presence of normal mucosa without abnormal mobility, pain, or sensitivity to percussion. Radiographic success: Associated with decrease in the size of radiolucency and the presence of bone regeneration. If the radiolucency remained stable without remarkable changes, the treatment was classified as suspected and required further observation. Treatment failure was classified into two degrees as (a) the radiolucency slightly increased in size, but it was separated from succeeding bud with adequate bone and (b) the radiolucency threatening the succeeding buds, so the tooth was extracted. Clinical and radiographic success data separated.
Calixto-Chanca et al., 2014 [37]	3-6.	48. Unreported sample calculation. 24 boys. 24 girls.	56 (molars and anterior). Unreported reason for treatment. Number of operators not reported (number of session not reported and chemical-mechanical technique). Number of evaluators not reported (blinding and calibration not reported).	Modified Guedes-Pinto paste (0.30 g of iodoform, 0.1 ml camphorated paramonochlorophenol, 0.25 ml sodium rifamycin SV, 5 mg prednisone and 0.30 mg zinc oxide).	CTZ (chloramphenicol 500 mg, tetracycline 500 mg, zinc oxide 1000 mg and a drop of eugenol).	15 days, 2 and 4.	Clinical success: Absence of espontaneous pain, change in gum color, submucosal abscess, mobility and fistula each (yes and no). Radiographic success: Periodontal ligament space (normal and widened), root resorption (physiological and pathological), interradicular area (apposition and resorption). Clinical and/or radiographic success data individual condition evaluated but not summarized.
Cassol et al., 2019 [38]	2-7.	23. Unreported sample calculation. 16 boys. 7 girls.	27. Caries and trauma. 1 operator (different number of session and chemical-mechanical technique). 2 evaluators (double-blinded and calibration realized).	Modified Guedes-Pinto paste (iodoform + camphorated parachlorophenol + ointment comprising 5.0 mg prednisolone acetate and 1.5 mg rifamycin).	Calen®/ZO paste (S.S.White Artigos Dentários Ltda., Rio de Janeiro,Brazil).	6,9 and 12.	Clinical success: Absence of signs or symptoms of infection, such as pain, swelling, fistula, or sensitivity to percussion. Radiographic success: Reduction in the size of the previous radiolucent area, or no new radiolucency. Clinical and radiographic success data separated.
Chen et al., 2017 [17]	5.88 (±1.27.)	155. Sample calculation. Distribution by gender not reported.	160 (molars). Caries. 1 operator (2 session and chemical-mechanical technique). 2 evaluators (double-blindedand calibration realized).	- Vitapex (Neo Dental Chemical Products Co. Ltd., Tokyo, Japan). - MPRCF® (0.21 g zinc oxide, 0.07 g eugenol, 0.18 g iodoform and 0.01 g calcium hy- droxide).	ZOE (0.42 g zinc oxide, 0.14 g eugenol).	6, 12 and 18.	Clinical success: Completely free of clinical signs and symptoms including pain, gingival abscesses, fistula openings, and abnormal mobility. Radiographic success: Abcense of pathologic external root resorption and no radiographic lesions. The radiographic examination also included resorption of excess extraradicularly extruded materials and filling material in the root canal, and direction of the succes- sor permanent molars. The overfilled material was described as non-resorbed, partly resorbed, or completely resorbed. The state of filling material in the root canal were divided into five levels: both root and filling no change; root no change but filling resorbed; root began resorption, filling resorbed at faster rate; root began re- sorption, filling resorbed at same rate (the distance from the apex in the radiograph to the bottom of the filling being less than 1 mm); root began resorption, filling resorbed at slower rate. Clinical and radiographic success data separated.
Divya et al., 2019 [36]	4-9 (6,25).	17. Unreported sample calculation. Distribution by gender not reported.	30 (molars). Caries. 1 operator (number of session not reported and chemical-mechanical technique). 1 evaluator (blinding and calibration not reported).	- Endoflas with propolis [Propolis liquid (Brazilian Green Bee Propolis Liquid Extract, Uniflora®) and Endoflas powder (Sanlor and Cia. S. En C. S., Colombia). The mixing ratio of Endoflas powder and Propolis liquid was 2:1].	- LSTR (3Mix) (Ciprofloxacin, metronidazole and minocycline) (1: 3: 3).	3, 6 and 12.	Clinical insuccess: Presence of spontaneous pain, swelling, Sinus tractand mobility and premature exfoliation. Radiographic insuccess: Whether any increase/decrease of periapical and furcation radiolucencies, de*via*tion in the path of eruption of succedaneous teeth, internal root resorption had decreased or increased following treatment, resorption of over‐pushed material, resorption of filling material with respect to root resorption. Clinical and radiographic success data separated.
Doneria et al., 2017 [27]	4-8.	64. Unreported sample calculation. Distribution by gender not reported.	64 (molars) Caries. 1 operator (number of session not reported and chemical-mechanical technique). 1 clinical evaluator (was the operator) and 2 radiographic evaluators independent (double-blinded and calibration realized).	Vitapex (Neo dental co., Tokyo, Japan).	- ZnO‐ozonated oil [ZnO powder (DPI, Mumbai, India) (0.2 g, arsenic free) and ozonated castor oil (0.007 cc Ozonil, Ozone Forum of India, Mumbai, India)]. 3Mix‑MP modified(ornidazole tablets 500 mg (Ornida, Aristo pharmaceuticals, India), ciprofloxacin tablets 500 mg (Ciplox®, Alchemist Ltd., India), and cefaclor tablets 250 mg (DistaclorTM DT, Baroque pharmaceuticals, India)(1: 1: 1).	1, 6, 12 and 18.	Clinical success: Absence of pain, presence of healthy soft tissue, and absence of abnormal mobility. Radiographic success: included static/reduction in size of intra‐radicular radiolucency, evidence of bone regeneration/continuity of lamina dura, and the absence of internal/external resorption. The treatment was judged to be successful when both clinical and radiographic criteria were fulfilled. Clinical and radiographic success data separated.
Goel et al., 2018 [12]	4-9.	120. Sample calculation. 77 boys. 43 girls.	120 (molars) Caries. 1 operator (number of session not reported and chemical-mechanical technique). Number of evaluators not reported (blinding and calibration not reported).	Endoflas (Sanlor laboratories).	- ZOE (Prevest Denpro). - ZnO-Aloe vera (not reported). - ZnO −10% NaF (preparado na Pharmacology Lab).	3, 6, 9 and 12	Clinical success: Absence of pain and swelling, no tenderness on percussion, absence of sinus or fistula and mobility and presence of healthy soft tissue (defined as the absence of swelling, redness, or sinus tract). Radiographic success: Absence of periapical or inter- radicular pathology, absence of internal or pathological external root resorption, reduction or no change in the size of interradicular radiolucencies, presence of evidence of bone regeneration and radiographic continuity of lamina dura. Clinical and radiographic success data separated.
Goinka et al. 2020 [20]	4-9.	50. Unreported sample calculation. Distribution by gender not reported.	51 (primary second molars). Teeth with chronic infection. Number of operators not reported (number of session not reported and chemical-mechanical technique). 3 evaluators (duble-blinded and calibration not report). The result was determined by an agreement of at least two observers.	Metapex (Meta Biomed/Korea).	ZOE paste (Vishal Dentocare Pvt., Ltd., Gujarat) = ZnO -Aloe vera gel (DPI, Mumbai, India, 0.2 g arsenic free)	3, 6 and 12.	Clinical success: Presence of normal mucosa without abnormal mobility, pain, or sensitivity to percussion. Radiographic success: Decrease in the size of radiolucency and the presence of bone regeneration. If the radiolucency remained stable without remarkable changes, the treatment was classified as suspected and required further observation. Treatment failure was classified into two degrees as (a) the radiolucency slightly increased in size, but it was separated from succeeding bud with adequate bone and (b) the radiolucency threatening the succeeding buds, so the tooth was extracted. Clinical and radiographic success data separated.
Kottapalli et al., 2019 [35]	4-10.	Not reported.	30 (molars). Caries. 1 operator (1 session and chemical-mechanical technique). Number of evaluators not reported (blinding and calibration not reported).	Endoflas FS (Sanlor and Cia. S. En C. S., Colombia).	Mixture of zinc oxide powder (DPI, Mumbai, India) and nanohydroxyapatite (Sigma – Aldrich, Saint Louis, USA) with saline.	3, 6 and 12.	Clinical success: Absence of pain, tenderness on percussion, swelling and mobility. Radiographic success: Resorption of overpushed material (if any), resorption of material with the physiologic root resorption, de*via*ted path of eruption of succedaneous teeth. Clinical and radiographic success data separated.
Moness et al., 2012 [21]	3-5.	80. Unreported sample calculation. Distribution by gender not reported.	160 (maxillary primary incisors). Caries. 1 operator (1 session and chemical-mechanical technique). Number of evaluators not reported (blinding and calibration not reported).	Zical (Zinc Oxide Eugenol, Bismuth Subcarbonate, Iodoform, Resins and Barium Sulphate, eugenol and excipients) (Prevest Denpro Limited).	- Apexcal (Calcium hydroxide, bismuth carbonate, polyethylene glycol, glycerine, water) (Ivoclar Vivadent Ag). - ZOE (El Gomhoriay)	1, 3, 6, 9 and 12.	Clinical evaluation criteria History of pain Loss of clinical crown/ coronal restoration, recurrent caries Sensitivity to percussion Signs of erythema or swelling in the surrounding gingival tissue and mucosa Presence of fistula / sinus in the surrounding gingival tissue and mucosa. Radiographic evaluation criteria Presence or absence of periapical radiolucency Presence or absence of pathological internal or external root resorption Presence or absence of widening of periodontal membrane space. Clinical and radiographic success data separated.
Mortazavi and Mesbahi, 2004 [10]	3-13.	58. Unreported sample calculation. 32 boys. 26 girls.	58 (molars and anterior). Caries and trauma. Number of operators not reported (2 sessions and chemical-mechanical technique). Number of evaluators not reported (blinding and calibration not reported).	Vitapex (Neo Dental Chemical Products Co., Ltd, Tokyo, Japan).	ZOE (Associated Dental Products Ltd).	3 and 10-16.	Clinical success: Without evidence of pain, fistula, intraoral swelling, extraoral swelling or abnormal mobility were completely free of clinical signs and symptoms at the follow-up. Radiographic success: Evidence of bone radiolucency in their preoperative radiographs demonstrated evidence of a reduction in the size of the radiolucent area at the follow-up, and without any evidence of a radiolucent area at the start of treatment showed no newly formed radiolu- cency after. Clinical and/or radiographic success data individual condition evaluated but not summarized.
Nakornchai et al., 2010 [28]	3-8.	37. Unreported sample calculation. Distribution by gender not reported.	50 (molars). Caries. Number of operators not reported (3Mix – 1session and Vitapex − 1 or 2 sessions with chemical-mechanical technique). 1 evaluator (single-blinded and calibration realized).	Vitapex (Neo Dental Chemical Products Co., Ltd, Tokyo, Japan).	3Mix-MP (met- ronidazole (FlagylÒ, Sanofi-Aventis, Thailand), ciprofloxacin (CiprobayÒ, Bayer, Germany), and minocycline (MinocinÒ, Wyeth, China) (1: 1: 1).	6 and 12.	Clinical insuccess: Absence of pain, gingival abscesses, fistula openings, or abnormal mobility were completely free of clinical signs and symptoms. Radiographic success: Static or reduced size of bifurcation ⁄ periapical radiolucency, no progression of pathologic external root resorption, no progression of internal root resorption, and no newly formed radiographic lesions. If calcified metamorphosis occurred, it was noted but not regarded as a treatment failure. Clinical and radiographic success data separated.
Ozalp et al., 2005 [14]	3-9.	76. Unreported sample calculation Distribution by gender not reported.	80 (molars). Unreported reason for treatment. 2 operators (1session or 2 session when not collaborative patient and chemical-mechanical technique). Number of evaluators not reported (single-blinded and calibration not reported).	Vitapex (Diadent Group Int. Inc., Burnaby, BC, Canada).	ZOE (Associated Dental Products Ltd., Purton, Swindon, Wiltshire, UK). Calcitur (Voco, Cuxhaven, Germany). Sealapex (Kerr, Sybron Dental Specialties, Inc, Salerno, Italy).	2, 4, 6, 8, 10, 12 and 18.	Clinical success: Absence of pain, gengival swelling, tenderness to percussion, abnormal mobility, fistula or abscess. Radiographic success: Absence of furcation or periapical radiolucency, non-continuity of lamina dura and pathologic root resorption. Clinical and radiographic success data separated.
Pandranki et al., 2018 [18]	4-9.	26. Unreported sample calculation. Distribution by gender not reported.	60 (not reported). Caries. 1 operator (number of session not reported and chemical-mechanical technique). 2 evaluators (double-blinded and calibration realized).	Endoflas (Sanlor and Cia. S. en C.S.,Colombia).	ZOE (Not reported).	3, 6, 12 and 24.	Clinical success: When tooth is asymptomatic (i.e. without pain, tenderness, abscess, and decrease or absence of mobility). Radiographic success: Reduction in the size of interradicular radiolucency or the size remaining same and also when no signs of internal or external pathological root resorption demonstrated. If the tooth was symptomatic, it was considered as clinical failure and increase in postoperative inter‐radicular radiolucency or development of new postoperative radiolucency was considered as a radiographic failure. Clinical and radiographic success data separated.
Pramila et al., 2016 [16]	4-9.	88. Unreported sample calculation. 40 girls. 48 boys.	111 (not reported). Caries. 1 operator (1 session and chemical-mechanical technique). 2 evaluators (double-blinded and calibration realized).	- RC Fill (Medensco DentalProducts, Mumbai, India). - Vitapex (Neo Dental International, Inc.).	ZOE (Pulpdent Corporation)	6, 12 and 30.	Clinical and radiographic criteria according to modified American Association of Endodontists. Clinical and radiographic success data separated.
Qadeer et al., 2016 [29]	3-8 (3.54 D*p* = 1,69).	100. Sample calculation. 54 girls 46 boys	100 (primary molars). Caries. Number of operators not reported (number sessions not reported). Number of evaluators not reported (blinding and calibration not reported).	Vitapex (Not reported).	3 Mix (Not reported).	6.	Radiographic success Absence of interradicular or periapical radiolucency. Radiographic success data separated.
Reddy and Fernandes, 1996 [13]	3-8.	26. Unreported sample calculation. 15 boys. 11 girls.	30 (posterior and anterior). Caries. Number of operators not reported (2 sessions and technique filling not reported). Number of evaluators not reported (blinding and calibration not reported).	Maisto (14 g zincoxide, 42 g iodoform, 2 g thymol, 3 g camphor and 0,5 g lanolin).	ZOE (Not reported).	3-6 and 9.	Clinical success Absence of pain, tenderness, mobility and health of tissues surrounding teeth. Radiographic sucess: Resorption of excesso material, interradicular radiolucencies and bone formation. Clinical and radiographic success data separated.
Rewal et al., 2014 [11]	4-9.	50. Unreported sample calculation. 28 boys. 22 girls.	50 (molars). Unreported reason for treatment. 1 operator (1 session and chemical-mechanical technique). 1 clinical evaluator and 2 radiographic evaluators (blinding not reported and calibration realized).	Endoflas (Sanlor and Cia. S. en C.S., Cali, Colombia),	ZOE (Not reported)	3, 6 and 9.	Clinical success: Absence of pain, redness, swelling, tenderness on percussion, and sinus or fistula. Radiographic success: Reduction in the size of interradicular radiolucency or the size remaining the same. Clinical and radiographic success data separated and summarized.
Subramaniam and Gilhortra, 2011 [19]	5-9.	Not report.	45 (molars). Caries. Number of operators not reported (1 session and chemical-mechanical technique). Number of evaluators not reported (blinding and calibration not reported).	- Endoflas FS (Sanlor e Cia. S.en C.S., Cali, Colombia). -Metapex (Meta Biomed Company Ltd.).	ZOE (Not reported).	3, 6, 12 and 18.	Clinical success: No gingival swelling/inflammation/redness. No sinus opening in the oral mucosa or purulent exudate expressed from the gingival margin. No abnormal mobility other than mobility due to normal exfoliation. Absence of pain on percussion/tenderness. Radiographic success: No evidence of extensive pathologic root resorption. Reduction or no change in pre-operative pathologic interradicular and/or periapical radiolucency. No evidence of development of new post-operative pathologic radiolucency involving the succedaneous tooth germ. Clinical and radiographic success data separated.
Trairatvorakul and Chunlasikaiwan, 2008 [15]	3-7 (5.6 +-1.2).	42. Unreported sample calculation. Distribution by gender not reported.	54 (lower molars). Caries. 1 operator (1 session and chemical-mechanical technique). 2 evaluators (blinding realized and calibration not reported).	Vitapex (Neo Dental, Tokyo, Japan).	Zinc Oxide (Thailaisart Co, Saraburi, Thailand) and Eugenol (Tien Yuan Chemical Co, Singapore).	6 and 12.	Clinical success: Absence of pain, presence of healthy soft tissue (defined as the absence of swelling, redness, or sinustract) and absence of abnormal mobility. Radiographic success: Continuity of the lamina dura, reduction in the size of any pathologic inter-radicular and/ or periapical radiolucencies, or evidence of bone regeneration. Two conditions were considered to require further observation before considering treatment as a failure or success: 1) absence of change or more discontinuity of lamina dura; 2) absence of change in size of radiolucent area. Clinical and radiographic success data separated and summarized.
Zacharczuk et al., 2019 [30]	G1: 6.15 (± 1.38) and G2: 6.3 (± 1.49).	Not report. Unreported sample calculation. Distribution by gender not reported.	46 (primary molars). Teeth diagnosed with pulp necrosis. 4 operators (1 session and chemical-mechanical technique) (trained and calibrated). Number of evaluators not reported (blinding and calibration not reported).	Maisto­Capurro paste: (equal parts by volume of calcium hydroxide and iodoform, with propylene glycol as carrier).	3Mix­MP (metronidazole, ciprofloxacin, minocycline in a ratio 1:1:1 by volume). Carriers (macrogol and propylene glycol) were also used in a ratio 1:1 by volume.	1, 3, 6, 12 and 18.	Clinical success: Absence of any of the following: pain or sensitivity to percussion and palpation, swelling, fistula and non­ physiological mobility. Radiographic success: Absence of internal or external non­physiological resorption, no progression or reduction of radiolucent periapical/interradicular lesion and evidence of bone regeneration. Clinical and radiographic success data separated.

ZOE: Zinc-Oxide and Eugenol. LSTR: Lesion Sterilization and Tissue Repair Therapy.

The number of patients included in the studies ranged from 27 to 120 children, and only three studies presented a sample calculation [12,17,29]. The age of the patients ranged between 3 and 13 years old. Thirteen studies reported exclusively pulpectomy resulting from complications of carious lesions [9,12,13,15–19,21,27,29,35,36], whereas other studies included deciduous teeth with pulpectomy associated with pulp lesions caused by carious lesions or trauma [10,38], chronic infection [20] or necrosis [30]. Finally, other studies did not clearly explain the clinical reason for root canal treatment [11,14,28,37].

Most studies included upper and lower molars [9,11,12,14,17,19,27–30,35,36], others only second molars [20], lower molars [15], anterior and posterior teeth [10,13,37,48], or only upper incisors [21]. Two studies did not report on the type of teeth included [16,18].

The root canal treatments were carried out by one operator [11,12,15,16,18,21,27,28,35,36,38], two [14] or four [30] operators or by an unreported number of operators [9,10,13,17,19,20,29,37]. The procedure was carried out in a single session [9,11,15,16,19,21,35] or in two sessions [10,13,17]. A further two studies used individualized protocols for deciding the number of root canal treatment sessions [14,38], while in the remaining nine studies the number of sessions was not reported [12,18,20,27–30,36,37].

In most studies, the chemomechanical technique was employed for the root canal treatment [9–12,14–21,27–30,35–38], while the LSTR therapy without instrumentation was employed in fewer studies [28–30,36,37]. Finally, one study did not clearly report on the technique employed [13].

Several iodoform-based filling materials had been used in the studies. Iodoform with calcium hydroxide was present in the commercial brands: Metapex [9,10,19,20] and Vitapex [14–17,27–29] and Maisto-Capurro paste [30]. Iodoform associated with zinc oxide, eugenol and calcium hydroxide was synthesized using all these components [17], without addition of chlorophenol [9], with addition of propolis [36], or as the commercial brand (Endoflas) [11,12,18,19,35]. One study used iodoform, zinc oxide and eugenol (RCFill) [16] and another study used iodoform, zinc oxide eugenol (ZOE), bismuth subcarbonate, resins, barium sulphate, eugenol and excipients (Zical) [21]. Two studies used different versions of the modified Guedes-Pinto paste [37,38], and finally one study used Maisto paste [13].

Several positive control groups (noniodoform-based filling materials) were found in the studies. ZOE was standard [9–21]; alone or in addition to other components such as chloramphenicol and tetracycline (CTZ) [37]. Other versions of zinc oxide (ZO) without eugenol were found, but with the addition of other active components such as calcium hydroxide [38], propolis [9], ozonized oil [27], *aloe vera* [12,20], sodium fluoride 10% [12] and nanohydroxyapatite [35]. Calcium hydroxide–based pastes were also used: Apexcal (calcium hydroxide, bismuth carbonate, polyethylene glycol, glycerine and water) [21], Sealapex [14] and Calcitur [14]. The material used in the LSTR technique was 3Mix paste (ciprofloxacin, metronidazole and minocycline) [28–30,36,37].

The number of follow-up sessions included a minimum of two [9,10,15,28,29] and a maximum of seven [14] clinical and/or radiographic appointments. The interval of between follow-ups sessions varied greatly from 15 days [37] to 30 months [16].

Clinical and/or radiographic evaluations were carried out by a single evaluator [15,19] or by two [16,38] or three evaluators [9,20]. Some studies reported that the evaluators were blinded or double-blinded for both the clinical and radiographic evaluations [9,14,16,20,38] others only for the clinical [28] or the radiographic evaluations [11,27]. Evaluators were previously calibrated to identify clinical and radiographic success in three studies [9,16,38] or only for radiographic success [11,27,28]. However, most of the studies did not report on the calibration process [10,12,13,15,17–19,21,29,30,35–37].

The clinical failure was assessed by the presence of: (1) pain [9–15,17–21,27,28,30,35–38], (2) mobility [9,10,12–15,17–20,27,28,30,35–37]; (3) tenderness on percussion [9,11,12,14,19–21,30,35,38]; (4) tenderness [13,18]; (5) fistula [10–12,14,17,18,21,28,30,37,38]; (6) swelling [10–12,14,15,19,21,30,35,36,38]; (7) sinus tract [11,12,15,19,21,36]; (8) redness [11,12,15,19]; (9) purulent exudate expressed from the gingival margin [19]; (10) premature exfoliation [36]; (11) loss of clinical crown/coronal restoration or recurrent caries [21]. Some researchers also noted the absence of normal mucosa [9,20,27,37] or the health of tissues surrounding the teeth [13,21]. In the present review, it was not possible to access the modified American Association of Endodontists criteria applied by Pramila et al. [16].

The radiographical failure was assessed by: (1) the presence of radiographic lesions [10,17]; (2) absence of reduction in the size of radiolucent area in the intra-radicular [27,36] or inter-radicular region [9,11–13,19,29,30,36–38], including the furcation [14,28,36,37] or periapical region [12,14,15,19,21,28–30]; (3) the absence of continuity of lamina dura [12,14,15,27]; (4) the absence of normal periodontal ligament space [21,37]; (5) the presence of external [12,17,21,27,28,30,38] or internal [12,21,27,28,30,38] root resorption [14,19,37]; (6) the presence of new radiolucency formed after of treatment [10,28]; (7) the presence of radiolucency involving the successor tooth germ [19]; (8) the presence of change in the direction of the successor tooth [17,35,36]; (9) the absence of bone regeneration [9,12,13,15,20,27,30]; (10) the absence of filling material in the root canal [17]; (11) the absence of extruded material extraradicularly [17]; (12) the absence of resorption of extravasated material [13,35,36] with physiologic root resorption [35,36].

### Risk of bias evaluation

Out of the 21 eligible studies, two [17,38] were evaluated as having a ‘low’ risk of bias, 13 as having an ‘unclear’ risk [9,11–15,18,19,27–29,35,36], and six studies as having a ‘high’ risk of bias [10,16,20,21,30,37] (Supplement 3).

### Meta-analysis

Two studies were excluded from the quantitative synthesis because it was not possible to extract the primary data needed to compare the outcomes studied [10,37]. Therefore, 19 studies remained for the meta-analysis of clinical or radiographic failures [9,11–21,27–30,35,36,38]. In the meta-analysis, LSRT was compared to iodoformed filling material. Thus, the result of LSRT group was not compared to conventional treatment subgroup without iodoform (0 failures in 20 cases in 6 and 12 months in clinical and radiographic evaluation).

**Figure 1.**
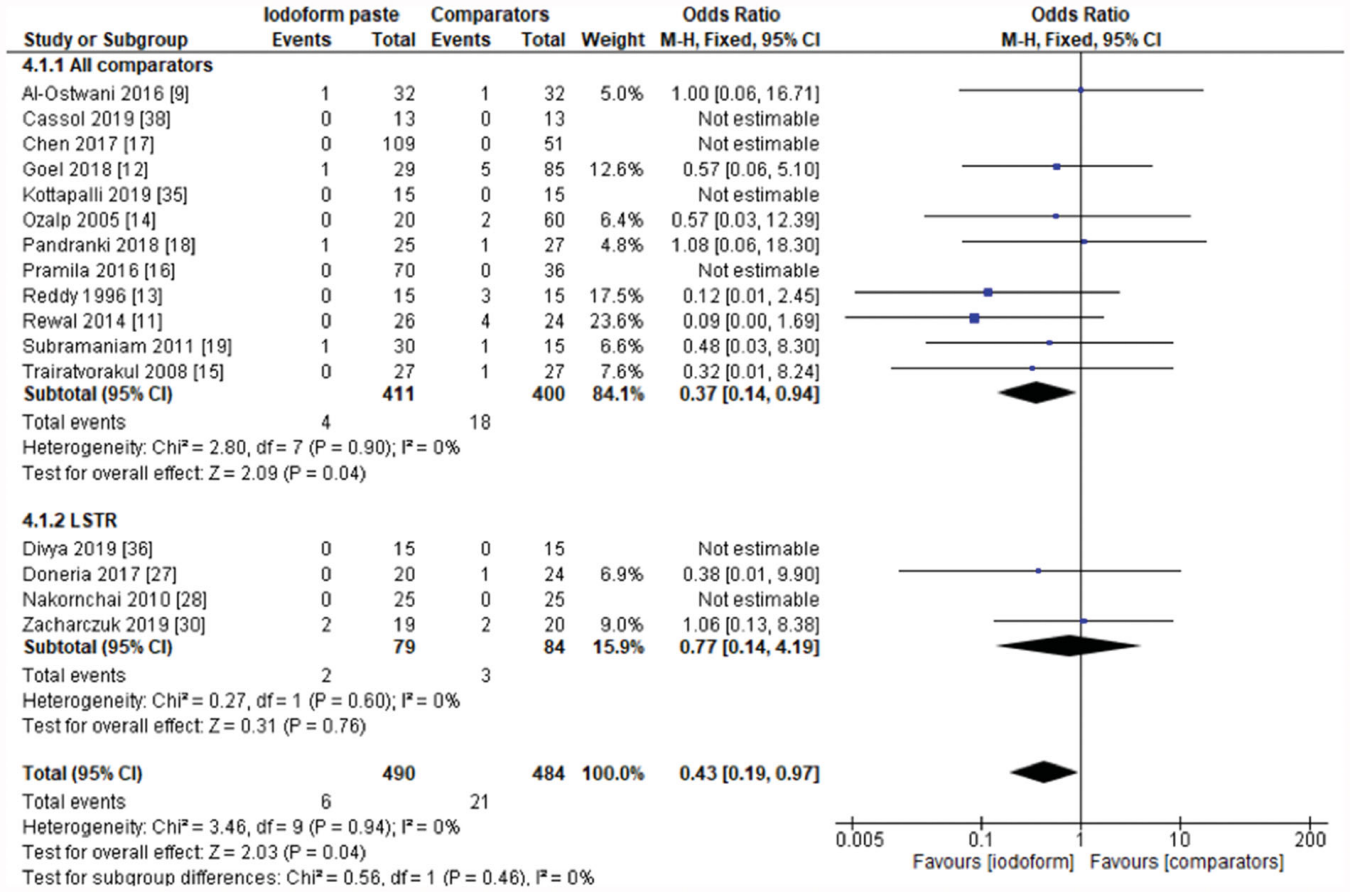
Forest-plot of the clinical evaluation after 6 months in deciduous teeth.

**Figure 2.**
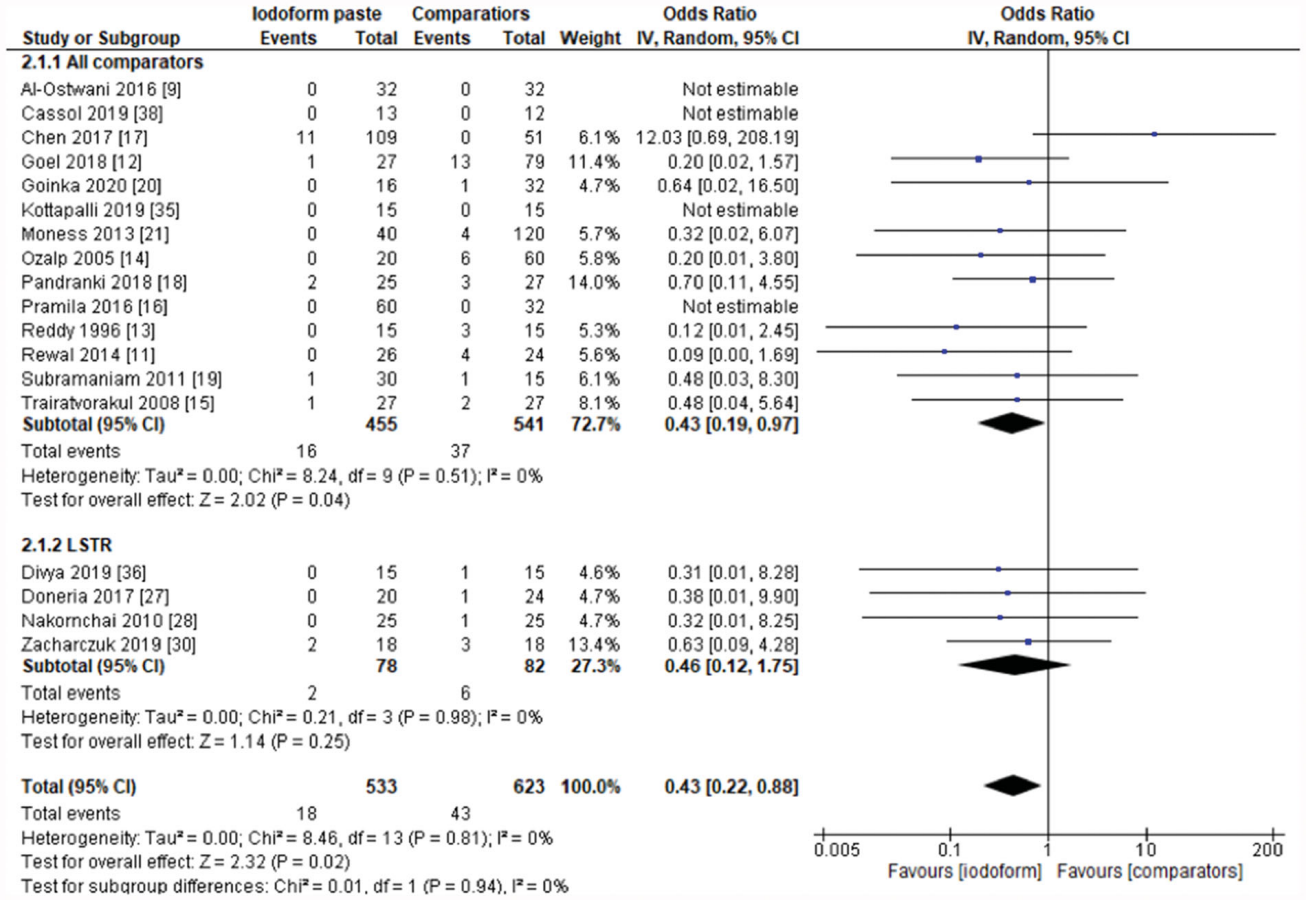
Forest-plot of the clinical evaluation after 9–12 months in deciduous teeth.

**Figure 3.**
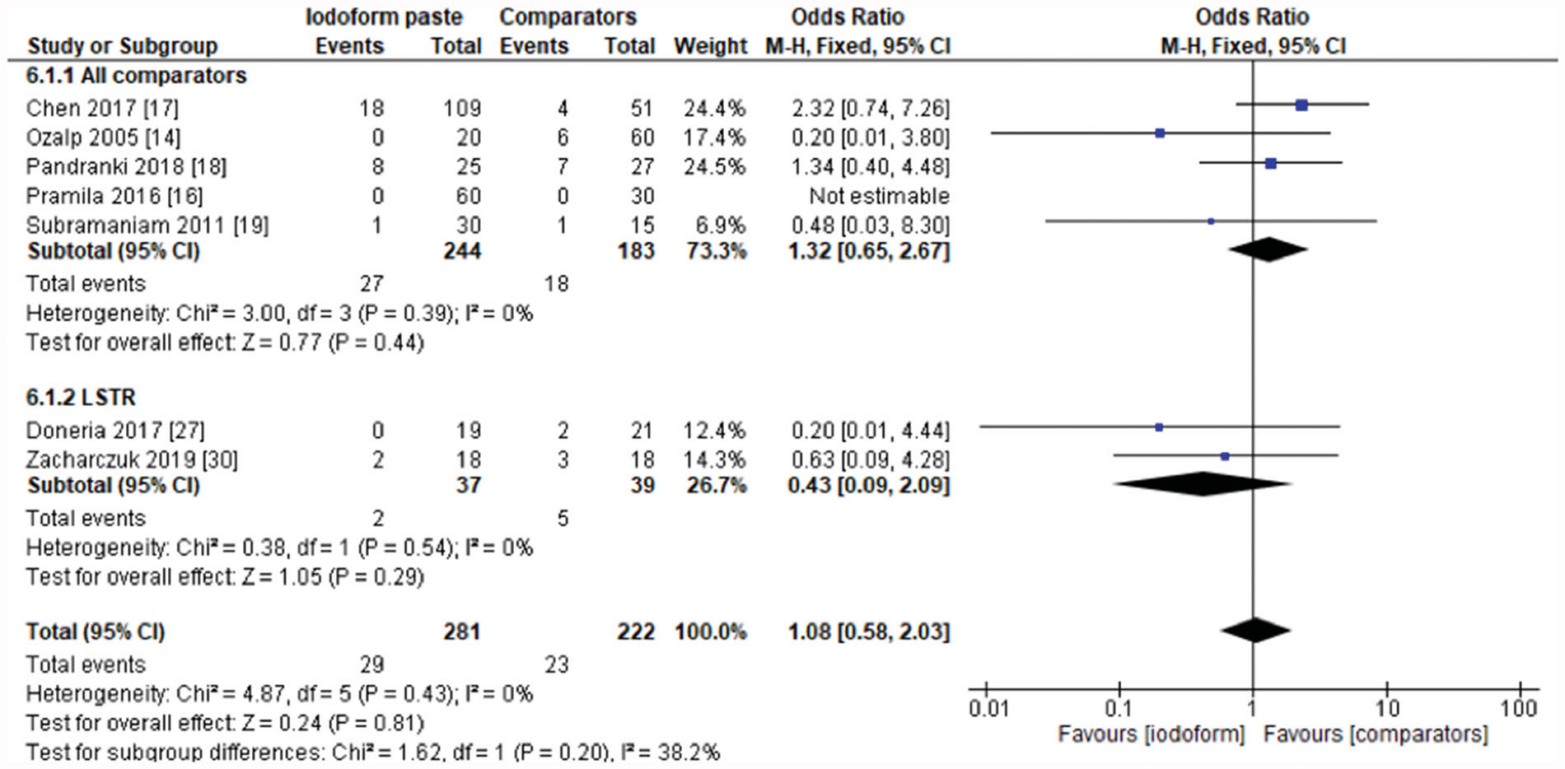
Forest-plot of the clinical evaluation after 18–30 months in deciduous teeth.

In the clinical evaluation, iodoform-based filling materials showed fewer failures at the 6 months (OR = 0.43, 95%CI: 0.19–0.97, *p* =.04) and 9–12 months follow-ups (OR = 0.46, 95%CI: 0.23–0.93, *p* = .03), but not at the 18–30 months follow-up (OR = 1.08, 95%CI: 0.58–2.03, *p* = .81). In the subgroup using chemomechanical preparation, the iodoform-based filling materials performed better than noniodoform-based materials both at the 6 months (OR = 0.37, 95%CI: 0.14–0.94, *p* = .04) and 9–12 months follow-ups (OR = 0.43, 95%CI: 0.19–0.97, *p* = .04), but not at the 18–30 months follow-up (OR = 1.32, 95%CI: 0.65–2.67, *p* =.44). In the subgroup using the LSTR technique, there was no difference between iodoform-based and noniodoform-based materials at any of the follow-ups (6 months [OR = 0.77, 95%CI: 0.14–4.19, *p* = 0.76], 9–12 months [OR = 0.57, 95%CI: 0.15–2.12, *p* = .2]), 18–30 months [OR = 0.43, 95%CI: 0.09–2.09, *p* = .44]). The results did not present heterogeneity for clinical failures (test χ^2^, *p* > .05, I^2^=0%) (Figures 1–3).

**Figure 4.**
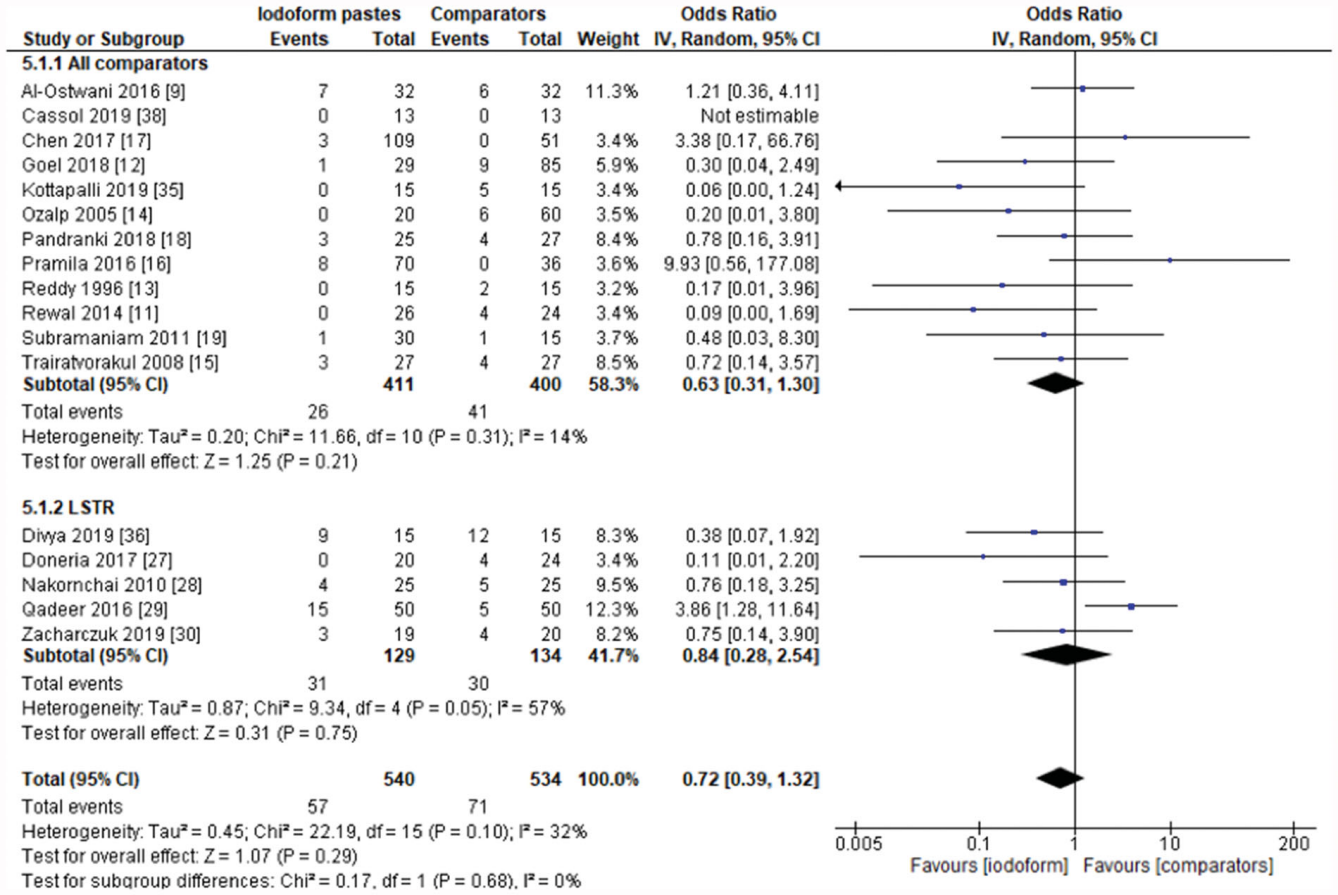
Forest-plot of the radiographic evaluation after 6 months in deciduous teeth.

**Figure 5.**
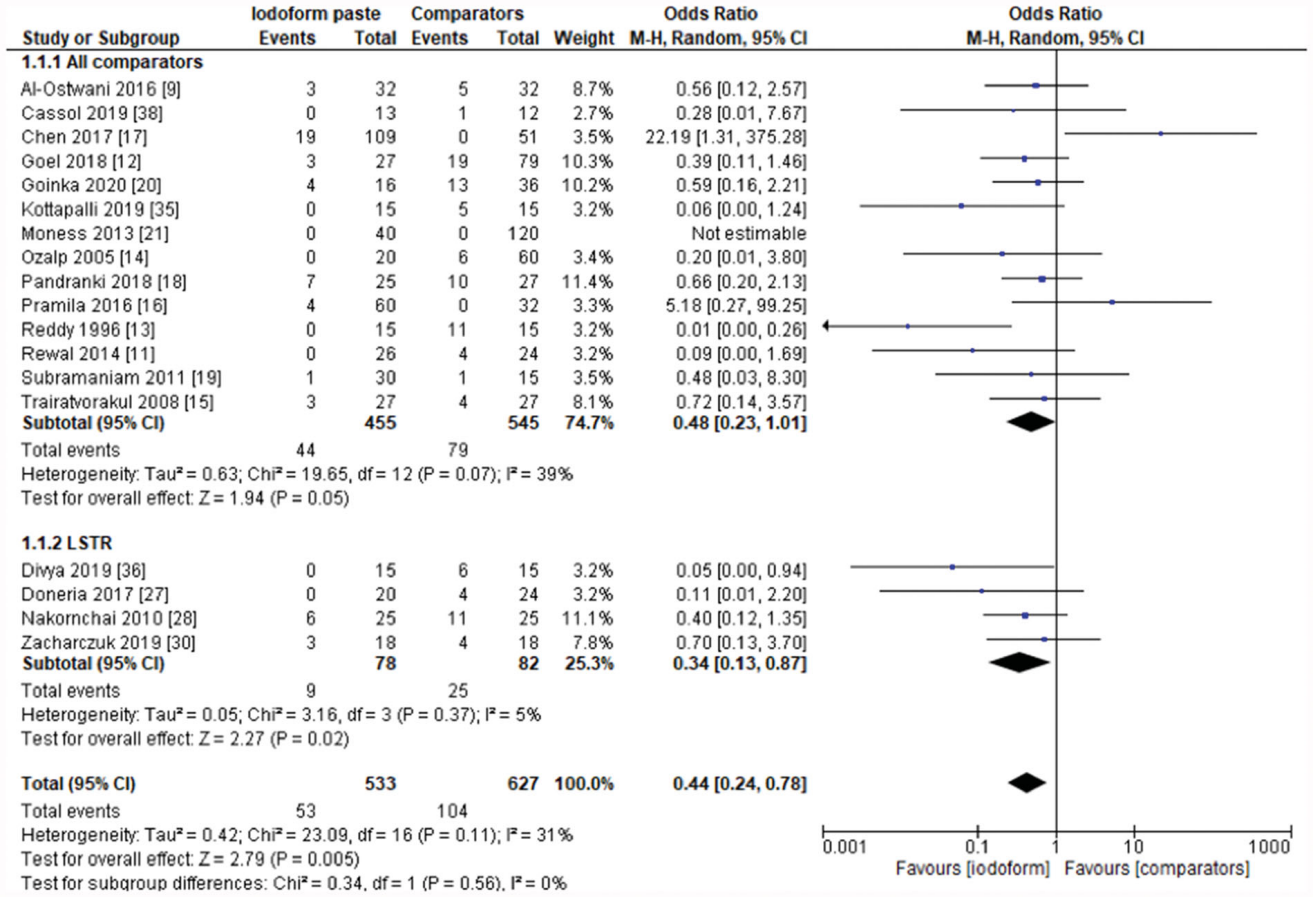
Forest-plot of the radiographic evaluation after 9–12 months in deciduous teeth.

**Figure 6.**
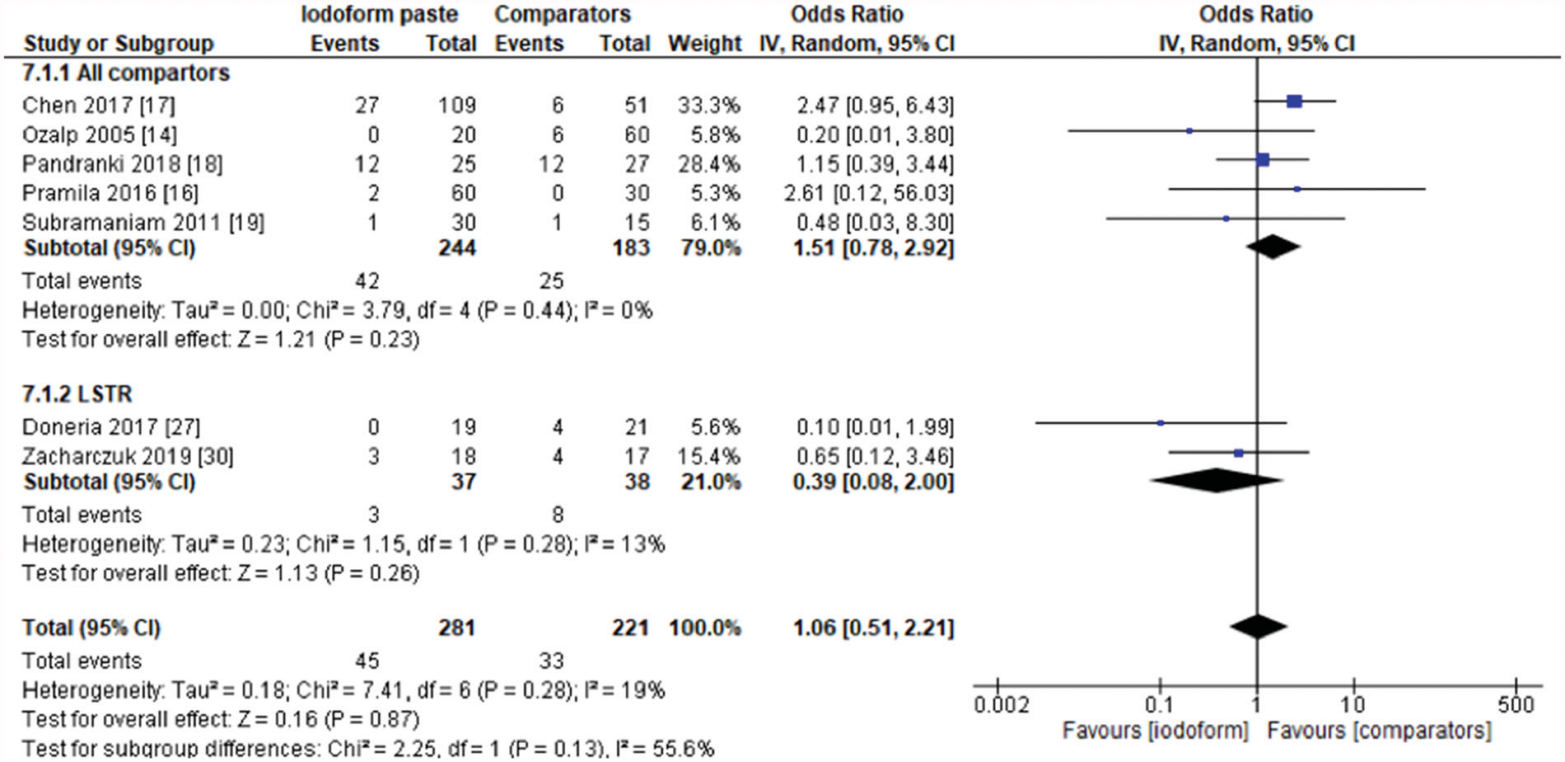
Forest-plot of the radiographic evaluation after 18–30 months in deciduous teeth.

In the radiographic evaluation, there was no statistical difference between iodoform-based and non-iodoform-based materials at the 6 months (OR = 0.72, 95%CI: 0.39–1.32, *p*=.29) and 18–30 months follow-ups (OR = 1.06, 95%CI: 0.51–2.21, *p* = .87), but fewer radiographic failures were detected for iodoform-based materials at the 9–12 months follow-up (OR = 0.49, 95%CI: 0.29–0.80, *p* = .005). In the chemomechanical preparation subgroup, there was no difference between iodoform-based and noniodoform-based materials at any of the follow-ups (6 months [OR = 0.63, 95%CI: 0.31–1.30, *p* = .21], 9–12 months [OR = 0.55, 95%CI: 0.30–1.02, *p* = .06], 18–30 months [OR = 1.51, 95%CI: 0.78–2.92, *p* = .23]). In the subgroup using the LSTR technique, there was no difference between iodoform-based and noniodoform-based materials at the 6 months (OR = 0.84;95%CI: 0.28–2.54, *p* = .75) and 18–30 months follow-ups (OR = 0.39; 95%CI: 0.08–2.00, *p* = .26), but iodoform-based materials showed lower failures rates at the 9–12 months follow-up (OR = 0.34; 95%CI: 0.13–0.87, *p* = .02). The results did not present heterogeneity (test χ^2^; *p* >.05; I^2^<32%), with the exception of the analysis of the subgroup using the LSTR technique at 6 months (test χ^2^; *p* = .05; I^2^ = 57%) (Figures 4–6).

### Assessment of the certainty of the evidence

The certainty of the evidence was assessed according to the evaluated outcomes: clinical and radiographic failure in the different follow-up periods (6, 9–12 and 18–30 months) and for the techniques for tooth preparation (chemomechanical and/or LSTR).

For the outcome ‘clinical failure’, the certainty of the evidence was graded as ‘very low’ for all follow-up periods, with the exception of the total (chemical-mechanical technique and LSTR) and LSTR subgroup analysis at the 6 and 9–12 months, which was rated ‘low’ (Table 2(A–C)).

**Table 2.** Summary of findings in Grading of Recommendations, Assessment, Development and Evaluation (GRADE) for clinical or radiographic evaluation at 6 months (A), 9-12 months (B) or 18-30 months (C) in deciduous teeth.

Outcomes	№ of participants (studies) Follow up	Certainty of the evidence (GRADE)	Relative effect (95% CI)	Anticipated absolute effects	
				Risk with non-iodoform-based filling materials	Risk difference with iodoform-based filling materials
A. Iodoform-based filling materials compared to non-iodoform-based filling materials in the root canal treatment of deciduous teeth with chemical-mechanical technique and/or LSTR at 6 months.					
Total: Chemical-mechanical technique and LSTR. Event: clinical failures. follow-up: 6 months.	974 (16 RCTs)	⨁⨁◯◯ LOW^a,b^	OR 0.43 (0.19 to 0.97)	43 per 1000	24 fewer per 1000 (35 fewer to 1 fewer)
Subgroup: Chemical- mechanical technique. Event: clinical failures. follow-up: 6 months.	811 (12 RCTs)	⨁⨁◯◯ LOW^a,b^	OR 0.37 (0.14 to 0.94)	45 per 1000	28 fewer per 1000 (38 fewer to 3 fewer)
Subgroup: LSTR. Event: clinical failures. follow-up: 6 months.	163 (4 RCTs)	⨁◯◯◯ VERY LOW^d,e^	OR 0.77 (0.14 to 4.19)	36 per 1000	8 fewer per 1000 (31 fewer to 99 more)
Total: Chemical-mechanical technique and LSTR. Event: radiographic failures follow-up: 6 months.	1014 (16 RCTs)	⨁◯◯◯ VERY LOW^d,e^	OR 0.78 (0.43 to 1.42)	51 per 1000	11 fewer per 1000 (28 fewer to 20 more)
Subgroup: Chemical- mechanical technique. Event: radiographic failures. follow-up: 6 months.	731 (12 RCTs)	⨁◯◯◯ VERY LOW^a,c,d^	OR 0.67 (0.32 to 1.43)	103 per 1000	32 fewer per 1000 (68 fewer to 38 more)
Subgroup: LSTR. Event: radiographic failures. Follow-up: 6 months.	283 (4 RCTs)	⨁◯◯◯ VERY LOW^d,e^	OR 0.92 (0.33 to 2.59)	195 per 1000	13 fewer per 1000 (121 fewer to 190 more)
B. Iodoform-based filling materials compared to non-iodoform-based filling materials in the root canal treatment of deciduous teeth with chemical-mechanical technique and/or LSTR at 9–12 months.					
Total: Chemical-mechanical technique and LSTR. Event: clinical failures. follow-up: 9–12 months.	1156 (18 RCTs)	⨁⨁◯◯ LOW ^a,b^	OR 0.46 (0.23 to 0.93)	69 per 1000	36 fewer per 1000 (52 fewer to 5 fewer)
Subgroup: Chemical- mechanical technique. Event: clinical failures. follow-up: 9–12 months.	996 (14 RCTs)	⨁⨁◯◯ LOW ^a,b^	OR 0.43 (0.19 to 0.97)	68 per 1000	38 fewer per 1000 (55 fewer to 2 fewer)
Subgroup: LSTR. Event: clinical failures. follow-up: 9–12 months.	160 (4 RCTs)	⨁◯◯◯ VERY LOW ^c,d^	OR 0.57 (0.15 to 2.12)	73 per 1000	30 fewer per 1000 (61 fewer to 70 more)
Total: Chemical-mechanical technique and LSTR. Event: radiographic failures. follow-up: 9–12 months.	1160 (18 RCTs)	⨁◯◯◯ VERY LOW ^a,b,e^	OR 0.43 (0.24 to 0.75)	166 per 1000	87 fewer per 1000 (120 fewer to 36 fewer)
Subgroup: Chemical- mechanical technique. Event: radiographic failures. follow-up: 9–12 months.	1000 (14 RCTs)	⨁◯◯◯ VERY LOW ^a,b,e^	OR 0.47 (0.23 to 0.96)	145 per 1000	71 fewer per 1000 (107 fewer to 5 fewer)
Subgroup: LSTR. Event: radiographic failures. Follow-up: 9–12 months.	160 (4 RCTs)	⨁⨁◯◯ LOW ^b,c^	OR 0.34 (0.13 to 0.87)	305 per 1000	175 fewer per 1000 (251 fewer to 29 fewer)
C. Iodoform-based filling materials compared to non-iodoform-based filling materials in the root canal treatment of deciduous teeth with chemical-mechanical technique and/or LSTR at 18-30 months.					
Total: Chemical-mechanical technique and LSTR. Event: clinical failures. follow-up: 18–30 months.	503 (7 RCTs)	⨁◯◯◯ VERY LOW ^a,d^	OR 1.08 (0.58 to 2.03)	104 per 1000	7 more per 1000 (41 fewer to 86 more)
Subgroup: Chemical- mechanical technique. Event: clinical failures. follow-up: 18–30 months.	427 (5 RCTs)	⨁◯◯◯ VERY LOW ^a,d^	OR 1.32 (0.65 to 2.67)	148 per 1000	38 more per 1000 (46 fewer to 169 more)
Subgroup: LSTR. Event: clinical failures. follow-up: 18–30 months.	76 (2 RCTs)	⨁◯◯◯ VERY LOW ^d,f^	OR 0.43 (0.09 to 2.09)	128 per 1000	69 fewer per 1000 (115 fewer to 107 more)
Total: Chemical-mechanical technique and LSTR. Event: radiographic failures follow-up: 18–30 months.	502 (7 RCTs)	⨁◯◯◯ VERY LOW ^a,d^	OR 1.06 (0.51 to 2.21)	149 per 1000	8 more per 1000 (67 fewer to 130 more)
Subgroup: Chemical- mechanical technique. Event: radiographic failures. follow-up: 18–30 months.	427 (5 RCTs)	⨁◯◯◯ VERY LOW ^a,d,g^	OR 1.51 (0.78 to 2.92)	137 per 1000	56 more per 1000 (27 fewer to 179 more)
Subgroup: LSTR. Event: radiographic failures. Follow-up: 18–30 months.	75 (2 RCTs)	⨁◯◯◯ VERY LOW ^d,f^	OR 0.39 (0.08 to 2.00)	211 per 1000	116 fewer per 1000 (190 fewer to 137 more)

LSTR: Lesion Sterilization and Tissue Repair Therapy.

The risk in the intervention group (and its 95% confidence interval) is based on the assumed risk in the comparison group and the relative effect of the intervention (and its 95% CI); CI: Confidence interval; OR: Odds ratio.

GRADE Working Group grades of evidence:

High certainty: We are very confident that the true effect lies close to that of the estimate of the effect.

Moderate certainty: We are moderately confident in the effect estimate: The true effect is likely to be close to the estimate of the effect, but there is a possibility that it is substantially different.

Low certainty: Our confidence in the effect estimate is limited: The true effect may be substantially different from the estimate of the effect.

Very low certainty: We have very little confidence in the effect estimate: The true effect is likely to be substantially different from the estimate of effect.

Explanations:

^a^Most of the included studies were judged to be at "unclear risk of bias".

^b^The optimal size effect was not achieved.

^c^There was heterogeneity that could not be explained.

^d^The optimal information sample was not met and the confidence interval crosses the threshold.

^e^All the included studies were judged to be at "unclear risk of bias".

^f^The included studies were at high and unclear risk of bias.

^g^There is inconsistency in the direction of the effect.

For the outcome ‘radiographic failure’, the certainty of the evidence was graded as ‘very low’ for all follow-up periods, with the exception of the LSTR subgroup at 9–12 months, which was rated ‘low’ (Table 2(A–C)).

## Discussion

This systematic review and meta-analysis compared iodoform-based and noniodoform-based filling materials for root canal treatment of deciduous teeth. Iodoform-based filling materials showed fewer clinical failures when compared to noniodoform-based materials after 6 and 9–12 months, and similar performance after 18–30 months. There were fewer radiographic failures of iodoform-based filling materials at the 9–12 months follow-up, but similar performance of the two groups of materials at the 6 and the 18–30 months follow-ups.

The similarity between the filling materials with and without iodoform at the 6 months radiographic evaluation may derive from the insufficient time to detect significant changes in bone neo-formation and lesion healing, particularly when the evaluation criteria were based on visual examination of the radiographs. More sensitive methods, like radiographic subtraction is known to better detect subtle changes in radiopacity that supersede human eye examination capacity [55]. Although they exhibit low heterogeneity, the loss of patients during follow-up was not explained by the authors, resulting in incomplete data outcome and this probably have influence in the results.

All systematic reviews published so far were inconclusive in identifying the best choice of filling material for primary tooth pulpectomy [4–7]. This systematic review was the first to detect significant differences between different filling materials. This result may indicate a positive effect on the clinical and radiographic outcomes of filling materials containing iodoform, an effect which can be explained by the high antimicrobial property of iodoform [8,10,34]. In general, the odds ratio for the treatment effect was obtained from an adequate sample size and a good number of studies were included. Even so, the certainty of the evidence was classified as ‘low’ or ‘very low’ for the evaluated outcomes. This limits our confidence, that is, the true effect may be substantially different from the estimate of the effect. The ideal scenario for analyzing the effect of iodoform-based filling material would be a study comparing the same filling material with and without iodoform.

Most studies used commercially available iodoform-based filling materials, such as Vitapex [14–17,27–29], Metapex [9,10,19,20], Endoflas [11,12,18,19,35], RC Fill [16], Zical [21], Maisto paste [13] or Maisto-Capurro paste [30]. Although many professionals choose premixed pastes due to ease of use, the professional must be aware of the material’s composition and follow manufacturer recommendations for a greater success rate. Only two studies [37,38] used an unmarketed, iodoform-based filling material in the form of a modified Guedes-Pinto paste. In this situation, the proportions of the components must be respected during handling to obtain the maximum benefit and the lowest risk of adverse effects. The resorption ability is an advantageous property of iodoform [8,10,34]. However, when iodoform-based filling materials are not used correctly, they have the disadvantage of compromising aesthetics through a brown-yellowish pigmentation of the dental crown [34]. To reduce pigmentation, during the performance of the clinical procedure, the operator must be careful cleaning of the pulp chamber after root canal obturation and before restoration.

The application of different eligibility criteria and different clinical protocols during endodontic treatment among the studies may have introduced some degree of variability and have influenced the rates of radiographic and clinical failures. Potentially influencing factors include: selection of the patient and tooth and the operator’s ability and preferred technique (manual, motorized files or LSTR). Also, the instrument size and the taper influence the flow of the root canal filling material and the removal of infectious radicular dentin [56]. Incomplete information about such bias factors as observed in the included studies make comparison between studies difficult. To facilitate the comparison between the techniques, subgroup analysis considering the different root canal techniques (chemomechanical root canal preparation and LSTR therapy) was performed.

The LSTR involves the use of a triple antibiotic mixture, i.e. a paste based on ciprofloxacin, metronidazole and minocycline (3Mix-MP) [3,28–30,36,37], aiming to disinfect the root canal system without any mechanical preparation. The performance of iodoform-based filling materials was similar in the chemomechanical technique and the LSTR therapy at the 6 and the 9–12 months clinical evaluations, and in the radiographic analysis at 6 months. The iodoform-based filling material was superior only in the radiographic comparison at the 9–12 months of follow-up. Another systematic review has previously demonstrated that the two treatments showed comparable outcomes, regardless of the follow-up period (6, 12 or 18 months) and type of evaluation (clinical or radiographical) with certainty of evidence ranging from very low to moderate [3].

It must be pointed out that the absence of difference in our metanalysis may be due to the limited number of studies and a small sample size that probably failed to identify differences between groups; also the certainty of evidence was graded as low and very low. Even so, it is worth noting that a simplified technique such LSTR can benefit professionals, as it will reduce clinical time and facilitate the treatment of non-collaborative children [3]. For this reason, future studies with the use of LSTR are encouraged and should compare not only the techniques used, but include test groups with and without iodoform even with the chemomechanical technique.

As recommended in systematic reviews about filling materials in deciduous teeth [3–7], there has been an increase in the number of randomized clinical trials in the last few years [9,11,12,17,20,21,27,29,30,35–38]. But it is relevant to point out that the quality of the trials was not improved sufficiently to strengthen the scientific evidence. This highlights the importance of best design and methodological accuracy of the primary studies, particularly regarding the randomization process and blinding of evaluators. Thus, further studies are still needed to increase the certainty of the evidence.

## Conclusion

Iodoform-based filling materials showed better clinical and radiographic performance when compared to noniodoform-based filling materials in the short term and similar performance in the long term. However, most of the studies showed unclear or high risk of bias and the overall certainty of the evidence ranged from low to very low. Therefore, new randomized clinical trials must be accomplished to corroborate this conclusion.

## Supplementary Material

Supplemental Material

Supplemental Material

Supplemental Material
