# Supplementary material for: Effectiveness of iodoform-based filling materials in root canal treatment of deciduous teeth: a systematic review and meta-analysis
Source: Biomater Investig Dent. 2022 May 19;9(1):52–74. doi: 10.1080/26415275.2022.2060232 (PMC9126566; doi:10.1080/26415275.2022.2060232)
Supplement: Supplemental Material [file IABO_A_2060232_SM2332.docx]

|  |  | | **Adequate sequence generation?** | | **Allocation concealment?** | | **Blinding?** | | **Incomplete outcome data addressed?** | | **Free of selective reporting?** | |  |
| --- | --- | --- | --- | --- | --- | --- | --- | --- | --- | --- | --- | --- | --- |
|  | | Al-Ostwani et al. 2016 [9] | |  | |  | |  | |  | |  | |
|  | Calixto-Chanca et al., 2014 [37] | |  | |  | |  | |  | |  | |  |
|  | | Cassol et al., 2019 [38] | |  | |  | |  | |  | |  | |
|  | | Chen et al., 2017 [17] | |  | |  | |  | |  | |  | |
|  | | Divya et al., 2019 [36] | |  | |  | |  | |  | |  | |
|  | | Doneria et al., 2017 [27] | |  | |  | |  | |  | |  | |
|  | | Goel et al., 2018 [12] | |  | |  | |  | |  | |  | |
|  | | Goinka et al., 2020 [20] | |  | |  | |  | |  | |  | |
|  | | Kottapalli et al., 2019 [35] | |  | |  | |  | |  | |  | |
|  | | Moness et al., 2013 [21] | |  | |  | |  | |  | |  | |
|  | Mortazavi e Mesbahi, 2004 [10] | |  | |  | |  | |  | |  | |  |
|  | Nakornchai et al., 2010 [28] | |  | |  | |  | |  | |  | |  |
|  | Ozalp et al., 2005 [14] | |  | |  | |  | |  | |  | |  |
|  | | Pandranki et al., 2018 [18] | |  | |  | |  | |  | |  | |
|  | | Pramila et al., 2016 [16] | |  | |  | |  | |  | |  | |
|  | | Qadeer et al., 2016 [29] | |  | |  | |  | |  | |  | |
|  | Reddy e Fernandes, 1996 [13] | |  | |  | |  | |  | |  | |  |
|  | Rewal et al., 2014 [11] | |  | |  | |  | |  | |  | |  |
|  | Subramaniam e Gilhortra, 2011 [19] | |  | |  | |  | |  | |  | |  |
|  | Trairatvorakul e Chunlasikaiwan, 2008 [15] | |  | |  | |  | |  | |  | |  |
|  | Zacharczuk et al., 2019 [30] | |  | |  | |  | |  | |  | |  |

Supplement 3. Summary of the risk of bias assessment according to the Cochrane Collaboration tool. Underlined authors provided extra information by e-mail to allow assessment of the risk of bias.
