# Supplementary material for: Effectiveness of iodoform-based filling materials in root canal treatment of deciduous teeth: a systematic review and meta-analysis
Source: Biomater Investig Dent. 2022 May 19;9(1):52–74. doi: 10.1080/26415275.2022.2060232 (PMC9126566; doi:10.1080/26415275.2022.2060232)
Supplement: Supplemental Material [file IABO_A_2060232_SM2329.docx]

Studies included in quantitative synthesis (meta-analysis)
(n = 19)

Studies included in qualitative synthesis

(n = 21)

Records identified through database searching (n = 6,079)

Records excluded after title screen (n = 5,031)

Records excluded after abstract screen (n = 66)

Cochrane:
(n = 1,149)

22/03/2021

Lilacs/BBO:
(n = 242)

22/03/2021

Web of Science:
(n = 2,631)

22/03/2021

Scopus:
(n = 1,149)

22/03/2021

PubMed:
(n = 908)

22/03/2021

Records screened
(n = 96)

Records after duplicates were removed with Endnote (n = 5,127)

## Identification

Additional records identified through other sources (n = 690)

## Screening

Studies excluded (n = 13):

- Non-randomic clinical trial (n = 1)
- Absence of iodoform group (n = 2)
- Absence of non-iodoform group (n = 5)
- Same previously selected data (n = 1)
- Same study with shorter follow-up time (n = 2)
- Published in Chinese language (n = 2)

## Elegibility

Full-text articles assessed for eligibility (n = 34)

Studies not included in the meta-analysis (n = 2):

- Not reporting the information (n = 2)

## Included

- (n = 1)

Não reportou a inf

Supplement 2. Flow diagram of the study in March 2021.
