# Supplementary material for: Effectiveness of iodoform-based filling materials in root canal treatment of deciduous teeth: a systematic review and meta-analysis
Source: Biomater Investig Dent. 2022 May 19;9(1):52–74. doi: 10.1080/26415275.2022.2060232 (PMC9126566; doi:10.1080/26415275.2022.2060232)
Supplement: Supplemental Material [file IABO_A_2060232_SM2326.docx]

Supplement 1. Study search strategy used in June 2019 with updated for March 2021.

| Medline/Pubmed |  |
| --- | --- |
| #1 (tooth, deciduous[MeSH Terms] OR "deciduous dentition"[Title/Abstract] OR "primary dentition"[Title/Abstract] OR "primary teeth"[Title] OR "primary tooth"[Title/Abstract] OR "first teeth"[Title/Abstract] OR "first tooth"[Title]) | #2 ((Pulpectomy[MeSH Terms] OR endodontics[MeSH Terms] OR "root canal preparation"[MeSH Terms] OR "root canal therapy"[MeSH Terms] OR endodontic[Title/Abstract] OR "iodoform"[Title/Abstract] OR iodoformed[Title/Abstract] OR "vitapex"[Title/Abstract] OR guedes pinto paste[Title/Abstract] OR endoflas[Title/Abstract] OR maisto[Title/Abstract] OR "Kri paste"[Supplementary Concept] OR "Kri paste"[Title/Abstract] OR "vitapex"[Title/Abstract]) NOT (Pulpotomy[MeSH Terms]) |
| Lilacs/BBO |  |
| #1 (tw:((mh:(‘‘tooth, deciduous’’)) OR (mh:(‘‘diente primario’’)) OR (mh:(‘‘dente decíduo’’)) OR (tw:(“decidous dentition”)) OR (tw:(“dentição decidua”)) OR (tw:(“dentición temporal”)) OR (tw:(“primary dentition”)) OR (tw:(“dentadura decidua”)) OR (tw:(“dentadura temporal”)) OR (tw:(“primary teeth”)) OR (tw:(“dentes decíduos”)) OR (tw:(“dientes primarios”)) OR (tw:(“primary tooth”)) OR (tw:(“dente decíduo”)) OR (tw:(“diente primario”)) OR (tw:(“first teeth”)) OR (tw:(“first tooth”))) | #2 (tw:((mh:(endodontics)) OR (mh:(endodontia)) OR (mh:(endodoncia)) OR (mh:(pulpectomy)) OR (mh:(pulpectomia)) OR (mh:(pulpectomía)) OR (mh:(''root canal therapy'')) OR (mh:(''tratamiento del conducto radicular’’ )) OR (mh:("tratamento do canal radicular’’ )) OR (tw:("endodontic treatment" )) OR (tw:("tratamento endodontico" )) OR (tw:("tratamiento endodontico" )) OR (tw:("endodontically treated teeth’’)) OR (tw:("dentes tratados endodonticamente")) OR (tw:("dientes tratados endodonticamente")) OR (tw:(iodoform)) OR (tw:(iodofórmio)) OR (tw:(iodoformium)) OR (tw:(''iodoform past'')) OR (tw:(''pasta iodoformada'')) OR (tw:(iodoformed)) OR (tw:(iodoformado)) OR (tw:(‘‘guedes pinto paste’’ )) OR (tw:(''pasta guedes-pinto'')) OR (tw:(''kri paste'')) OR (tw:(''pasta kri'')) OR (tw:(vitapex)) OR (tw:(''maisto’s paste’’)) OR (tw:(''pasta maisto'')) OR (tw:(''endoflas'')) OR (tw:(''root filling paste'')) OR (tw:(''pastas de obturação de canal'')) OR (tw:(''carpetas obturadoras del canal'')) OR (tw:(''antibiotic paste'')) OR (tw:(''pasta de antibiótica'')) OR (tw:(''antimicrobial paste'')) OR (tw:(''pasta antimicrobiana'')))) |
| ISI Web of Science |  |
| #1 TS=(''tooth deciduous'') OR TS=(''deciduous dentition'') OR TS=(''primary dentition'') OR TS=(''primary t??th'') OR TS=(''first t??th'') | #2 TÓPICO: (Pulpect*) OR TS=(Endodontic) OR TS=(''root canal therap*'') OR TS=(''endodontic treatment'') OR TS=(''endodontically treated t??th'') OR TS=(''root canal preparation'') OR TS=(''root canal therapy'') OR TS=(''root filling paste'') OR TS=(''antibiotic paste'') OR TS=(''antimicrobial paste'') OR TS=(iodoform*) OR TS=(vitapex) OR TS=(''guedes pinto paste'') OR TS=(maisto) OR TS=(endoflas) OR TS=(''Kri paste'') |
| Cochrane Library |  |
| #1 (tooth deciduous):ti,ab,kw OR (deciduous dentition):ti,ab,kw OR (primary dentition):ti,ab,kw OR (primary t??th):ti,ab,kw OR (first t??th):ti,ab,kw (Word variations have been searched) | #2 (Pulpect*):ti,ab,kw OR (Endodontic):ti,ab,kw OR (root canal therap*):ti,ab,kw OR (endodontic treatment):ti,ab,kw OR (endodontic* treated t??th):ti,ab,kw OR (root canal):ti,ab,kw OR (preparation root canal):ti,ab,kw OR (therapy root filling):ti,ab,kw OR (paste antibiotic):ti,ab,kw OR (paste antimicrobial):ti,ab,kw OR (paste iodoform*):ti,ab,kw OR (vitapex):ti,ab,kw OR (guedes pinto paste):ti,ab,kw OR (endoflas):ti,ab,kw OR (maisto):ti,ab,kw OR (Kri paste):ti,ab,kw |
| Scopus |  |
| #1 ( TITLE-ABS-KEY ( ''tooth,deciduous'' ) OR TITLE-ABS-KEY ( ''deciduous AND dentition'' ) OR TITLE-ABS-KEY ( ''primary AND dentition'' ) OR TITLE-ABS-KEY ( ''primary AND t??th'' ) OR TITLE-ABS-KEY ( ''first AND t??th'' ) | #2 (TITLE-ABS-KEY ( pulpect* ) OR TITLE-ABS-KEY ( endodontic ) OR TITLE-ABS-KEY ( ''root AND canal AND therap*'' ) OR TITLE-ABS-KEY ( ''endodontic AND treatment'' ) OR TITLE-ABS-KEY ( ''endodontically AND treated AND t??th'' ) OR TITLE-ABS-KEY ( ''root AND canal AND preparation'' ) OR TITLE-ABS-KEY ( ''root AND canal AND therapy'' ) OR TITLE-ABS-KEY ( ''root AND filling AND paste'' ) OR TITLE-ABS-KEY ( ''antibiotic AND paste'' ) OR TITLE-ABS-KEY ( ''antimicrobial AND paste'' ) OR TITLE-ABS-KEY ( iodoform* ) OR TITLE-ABS-KEY ( vitapex ) OR TITLE-ABS-KEY ( ''guedes AND pinto AND paste'' ) OR TITLE-ABS-KEY ( endoflas ) OR TITLE-ABS-KEY ( maisto ) OR TITLE-ABS-KEY ( ''kri AND paste'' )) |
